# Therapeutic Efficacy of Soy-Derived Bioactives: A Systematic Review of Nutritional Potency, Bioactive Therapeutics, and Clinical Biomarker Modulation

**DOI:** 10.3390/foods14193447

**Published:** 2025-10-09

**Authors:** Zara Fatima, Nizwa Itrat, Beenish Israr, Abdul Momin Rizwan Ahmad

**Affiliations:** 1 Institute of Home Sciences, Faculty of Food Nutrition and Home Sciences, University of Agriculture, Faisalabad 38000, Pakistan; zarafatima225@gmail.com (Z.F.); nizwa.itrat@uaf.edu.pk (N.I.); 2 Department of Human Nutrition & Dietetics, NUST School of Health Sciences, National University of Sciences & Technology (NUST), Sector H-12, Islamabad 44000, Pakistan; 3 Department of Health Sciences, University of York, York YO10 5NH, UK

**Keywords:** soybean, chronic diseases, anti-inflammatory, protein energy malnutrition, therapeutic role, sustainable, bioactive compounds, fortification, fermentation

## Abstract

Soybeans (*Glycine max*) are nutrient-dense legumes and a high-quality plant-based protein source containing all essential amino acids. With a protein content of 36–40%, soy surpasses many other plant-derived proteins in nutritional value. Its bioactive components, particularly peptides and isoflavones, contribute to reducing inflammation, oxidative stress, and the risk of chronic diseases. In undernourished regions such as Pakistan, where protein-energy malnutrition is prevalent among women and children, soy offers a sustainable and cost-effective nutritional intervention. This review synthesizes findings from biochemical analyses, nutritional profiling, and clinical trials evaluating the impact of soybean protein and its bioactive compounds on growth, metabolic health, immune function, and disease prevention. Emphasis was placed on studies relevant to food-insecure populations and technological innovations enhancing soy product bioavailability. Soy protein has been shown to have positive effects on hormonal regulation, cardiovascular health, cognitive function, and immune support. Technological approaches such as fortification and fermentation improve nutritional bioavailability and sensory acceptance. The integration of soy into local diets enhanced nutritional adequacy, promoted environmental sustainability, and aligned with Sustainable Development Goals. Soybeans represent a sustainable, nutrient-rich solution to combat protein-energy malnutrition in vulnerable communities. Their high-quality protein profile, therapeutic properties, and adaptability to local food systems make them an effective strategy for improving public health and supporting environmental resilience.

## 1. Introduction

Soybean (*Glycine max*) stands out as a sustainable, nutrient-dense crop with significant potential to address global nutritional deficiencies while reducing the environmental burdens associated with conventional animal protein sources [1]. It is one of the most extensively cultivated and consumed legumes globally, valued for its high protein content, versatile applications, and significant role in the food and feed industries [2]. Globally, production of soybeans reached approximately 353 million metric tons, with the leading producers being the United States, Brazil, and Argentina. These three countries account for over 75% of the global soybean output, demonstrating the crop’s geographical concentration in the Americas. The cultivation area worldwide covers nearly 147 million hectares, making soybeans the fourth most widely grown crop after maize, wheat, and rice [3].

Soybean consumption is likewise increasing, driven by rising demands for plant-based protein, animal feed, edible oil, and industrial products [4]. Human consumption is in the form of soy-based foods such as tofu, soy milk, tempeh, soybean oil, soymeal, and textured soy protein [5]. Asia, especially China, is the world’s largest consumer and importer of soybeans, accounting for more than 60% of global imports to support its growing demand for feed and food-grade soy [6]. Additionally, the rise in vegetarian, vegan, and flexitarian diets in North America and Europe is accelerating the use of soy in meat and dairy alternatives, further boosting its global consumption [7]. These statistics underscore the strategic significance of soybeans in promoting food security, fostering economic growth, and promoting sustainable agriculture globally.

Soybean (*Glycine max* L.) is globally recognized as one of the most valuable and versatile leguminous crops due to its exceptional biochemical composition, economic significance, and profound nutritional and therapeutic properties [8]. Rich in high-quality plant protein (approximately 36–40%), essential amino acids, unsaturated fatty acids, dietary fiber, vitamins (notably folate and vitamin K), minerals (such as iron, calcium, magnesium), and bioactive phytochemicals like isoflavones, soybeans contribute significantly to human health and nutritional security [9].

From a nutritional perspective, soy protein is a complete protein in terms of digestibility and biological value, like animal proteins. Its incorporation into human diets has been associated with a broad array of health benefits, including reduced incidence of cardiovascular diseases, certain cancers, type 2 diabetes, osteoporosis, and even menopausal symptoms [10]. The soybean’s phytoestrogens, most notably genistein and daidzein, exhibit estrogen-like activity, modifying endocrine function and inflammation [11]. In addition, fermented products like miso, tempeh, and natto are good sources of probiotics that balance gut microbiota and boost immunity. Economically, soybeans dominate the world oilseed market and are a major agricultural export for the United States, Brazil, Argentina, and China [12]. Eating soy foods, like tofu, edamame, and soy milk in a normal diet, is safe for a healthy person. They do not have enough concentrations of phytoestrogens in those foods to produce toxic effects. The upper limit of the recommended dietary allowance (RDA) for the toxic effects of phytoestrogens in the human body is really a nonexistent value, for normal oral consumption of soy as food will not suffice to reach levels of toxicity in that regard. Studies reporting adverse effects typically do so under very high non-dietary intake, such as from concentrated supplements, and even the latter’s results are often hotly debated. A large body of evidence on this topic has repeatedly shown that moderate consumption of soy is not only safe but potentially beneficial for health by reducing the risk of cardiovascular disease and some types of cancer [13,14].

The increasing demand for plant-based protein sources, sustainable agriculture, and functional foods has further accelerated soybean production and innovation in soy-derived products. Moreover, advancements in biotechnology, such as genetically modified (GM) soybean varieties, have improved resistance to pests, environmental stressors, and crop yield [15].

Soybeans are a miracle crop for treating protein-energy malnutrition due to their great potential for use in regions lacking access to animal protein [16]. It is also a nitrogen-fixing plant that enhances soil fertility, thereby contributing to the sustainability of agriculture [17]. World problems of health, sustainability, and food system resilience can, therefore, have soybeans as part of their solutions because of the different food, medicinal, and industrial uses of the beans [18]. This review uses only recent high-quality studies (2018–2025) on soybean’s health effects, addressing outdated data issues. However, it leaves a gap in comparing these findings with earlier research to assess long-term trends and consistency.

## 2. Materials and Methods

For this review, we have selected research papers from the most trusted and authentic databases, Google Scholar, PubMed, Research Gate, etc.

Search Strategy: A broad search was made in the literature through PubMed, Scopus, and the Web of Science databases to identify randomized controlled trials (RCTs) and clinical studies of the effects of consumption of soy on human health outcomes from January 2018 to March 2025. Search terms included combinations of keywords such as “soy,” “soy protein,” “isoflavones,” “fermented soy,” “human trial,” “clinical study,” and “health outcomes.” No language restrictions were imposed.

**Figure 1:**
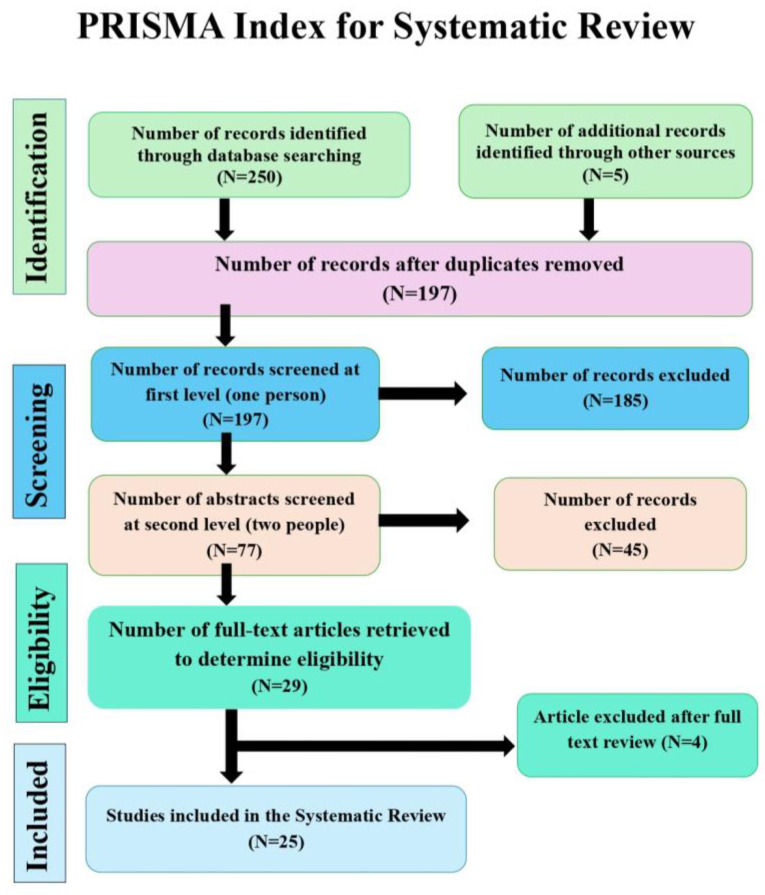
PRISMA flow diagram illustrating study selection process.

Eligibility Criteria
  • Included human clinical trials or RCTs.
  • Investigated soy or soy-derived products (e.g., tofu, tempeh, soy milk, or soy protein isolate).
  • Reported outcome measures related to metabolic health, markers of cardiovascular function, body composition, hormonal effects, or cognitive function.
  • Published from 2018 to 2025.
  • Exclusion criteria included: Animal or in vitro studies. Studies without a control group. Trials lacking sufficient data for extraction.
Selection of the Studies
  • The titles and abstracts of the identified studies were independently screened for relevance by two independent reviewers. Full texts of the studies deemed potentially eligible were retrieved and examined against the inclusion criteria. Disagreements were resolved through discussion or consultation with a third reviewer.
Data Extraction
  • Data were extracted using a standardized form, which included:
  • Author(s), year, country
  • Study design and duration
  • Participant characteristics (age, sex, health status)
Quality Assessment
  • The methodological quality of the included studies was assessed using the Cochrane Risk of Bias Tool for RCTs. For each examined criterion such as randomization, blinding, and outcome reporting, studies were classified as low, unclear, or high risk of bias (Figure 1, Table 1).

**Table 1:** Systematic review table: soy and human health (2018–2025).

Sr. No	Title of Study (Human Trials)	Authors	Year	Focus Area	Study Time	Country of Study	Reference
1.	A controlled randomized trial of metabolic syndrome, Consumption of soy milk enriched the plant sterols in a healthy dietary pattern lowers blood pressure in adults	Xu et al.	2025	Effect of sterol-enriched soy milk on metabolic and blood pressure in adults	Not specified	Singapore	[19]
2.	Evaluation of the Safety Profile of Symbiota^®^, a Fermented Soybean-Based Formulation Through Preclinical Tests and a Human Randomized Controlled Trial for Functional Food and Supplement Use	Hung et al.	2024	Safety, tolerability, and clinical effects of consuming the fermented soybean solution (Symbiota^®^)	Not specified	Taiwan	[20]
3.	Impact of Soy Extract-Based Nutraceuticals on Health Parameters in Postmenopausal Women: Findings from a Placebo-Controlled Randomized Trial of Double-Blind	Takuathung et al.	2024	Soy extract nutraceutical benefits: skin aging, bone health, cholesterol, and general health in postmenopausal women	12 weeks (typical duration for such interventions)	Thailand	[21]
4.	Assessing the Effectiveness of Doenjang, a Traditional Korean Fermented Food, in Alleviating Menopausal Symptoms and Managing Obesity: Findings from a Double-Blind Randomized Clinical Study	Han et al.	2024	Menopausal symptom relief, anti-obesity effect of fermented soy (Doenjang)	Not specified (likely 8–12 weeks typical)	Republic of Korea	[22]
5.	A Randomized Controlled Study on the Influence of Soy-Derived Isoflavones on Metabolic Parameters in Non-Alcoholic Fatty Liver Disease Patients	Neshatbini et al.	2024	Soy isoflavones’ impact on metabolic status in NAFLD patients	12 weeks	Iran	[23]
6.	Clinical Investigation of the Effects of Equol and Resveratrol on Bone Metabolism Indicators in Postmenopausal Women	Corbi et al.	2023	Evaluation of the effects of equol and resveratrol supplementation on bone turnover biomarkers in postmenopausal women	Not specified	Italy	[24]
7.	Assessment of the Safety Profile and Tolerance of Whole Soybean Consumption in Obese Elderly Individuals: A Gradual Dose-Increase Clinical Study	Rebello et al.	2023	Safety, tolerability, gastrointestinal responses, and biomarkers after escalating soybean flour intake	8 weeks	United States	[25]
8.	Impact of Probiotic-Enriched Soymilk Supplementation on Cardiovascular Risk Indicators in Individuals with Type 2 Diabetes	Hasanpour et al.	2023	Cardiovascular health in T2DM patients	8 weeks	Iran	[26]
9.	Clinical Insights into the Role of Soybean in Personalized Nutrition and Precision Healthcare	Kang et al.	2023	Soybeans, clinical benefits, precision medicine	Not specified	Republic of Korea	[27]
10.	An Overview of the Impact of Soy Consumption on Digestive System Health	Belobrajdic et al.	2023	Soy and gut health	Not specified	Australia	[28]
11.	A Three-Month Double-Blind Randomized Controlled Feeding Study Comparing the Impact of Soybean Oil (High in n-6 PUFA), Olive Oil (High in MUFA), and Camellia Seed Oil on Body Weight and Cardiometabolic Health in Chinese Women	Wu et al.	2022	Assessed the comparative effects of different dietary oils (soybean oil vs. olive oil vs. camellia seed oil) on body weight, lipid profile, glucose levels, and cardiometabolic risk factors in women	3 Months	China	[29]
12.	Cholesterol-Lowering Impact of a Non-Probiotic Fermented Soy Formula: Findings from a Randomized Crossover Clinical Trial	Jung et al.	2021	Cardiovascular health, lipid profiles (total cholesterol, LDL-C, HDL-C, ApoB/ApoA1), and isoflavone bioavailability	Not specified	United States	[30]
13.	Extended Intake of Soy Nuts Enhances Brain Blood Circulation and Motor Function: Evidence from a Randomized Controlled Crossover Study in Elderly Adults	Kleinloog et al.	2021	Cognitive health, cerebral blood flow, and response time	Not specified	The Netherlands and the USA	[31]
14.	Black Soybean Consumption Enhances Blood Vessel Health and Regulates Blood Pressure: Findings from a Human Randomized, Placebo-Controlled Crossover Study	Yamashita et al.	2020	Cardiovascular health, vascular function, blood pressure, nitric oxide, oxidative stress	4 weeks	Japan	[32]
15.	Impact of Whole Soy and Daidzein Isoflavone Supplementation on Bone Metabolism and Inflammation: A Six-Month Double-Blind Randomized Controlled Trial in Equol-Producing Postmenopausal Chinese Women	Liu et al.	2020	Bone health, inflammation, soy isoflavones	6 months	China	[33]
16.	Intake of Q-CAN Plus, a Fermented Soy-Based Drink, Enhances Serum Lipid Profile and Modulates Cytokine Levels	Arumuga et al.	2020	Investigating the effects of fermented soy beverage (Q-CAN Plus) on serum cholesterol and cytokine profiles	4 weeks (intervention period in a randomized, placebo-controlled design)	United States	[34]
17.	A Randomized Clinical Investigation into the Influence of Lunasin Extracted from Soybeans on Cardiometabolic Health Parameters	Haddad et al.	2020	Effects of soy peptide (lunasin) on lipid profiles, blood pressure, and inflammatory biomarkers in at-risk adults	Not specified	United States	[35]
18.	Impact of Phytosterol-Enriched Soy-Based Beverage on Blood Lipid Reduction	Chau et al.	2020	Lipid profile management in humans	8 weeks	Hong Kong/China	[36]
19.	Effectiveness and Safety of Fermented Soy Supplement DW2009 in Mild Cognitive Impairment: A 12-Week Randomized Controlled Trial	Hwang et al.	2019	Cognitive function and safety evaluation of a fermented soy supplement in older adults	12 weeks	Republic of Korea	[37]
20.	Soy Intake and Its Impact on Clinical and Metabolic Health in Older Women with Metabolic Syndrome: A Randomized Trial	Bakhtiari et al.	2019	Soy supplementation, metabolic syndrome	12 weeks	Iran	[38]
21.	Impact of Probiotic Soy Milk on Renal Function in Diabetic Nephropathy	Miraghajan et al.	2019	Probiotic soy milk, renal function in diabetics	8 weeks	Iran	[39]
22.	Assessment of Soy Milk Consumption on Metabolic Parameters in Patients Diagnosed with NAFLD: A Randomized Controlled Trial	Eslami et al.	2019	Soy milk, NAFLD, metabolic health	8 weeks	Iran	[40]
23.	Influence of S-equol and Soy-Derived Isoflavones on Cardiovascular and Neurological Health	Sekikawa et al.	2019	Soy isoflavones, cardiovascular and cognitive health	Not specified	Japan and USA	[41]
24.	Replacing Red Meat with Soybeans, but Not Other Legumes, Reduces Inflammatory Markers in Type 2 Diabetic Patients: Evidence from a Randomized Clinical Study	Hematdar et al.	2018	Soy consumption and inflammation in T2DM	Not specified	Iran	[42]

## 3. Phytochemical Composition of Soybean: A Bioactive Treasure

**Figure 2:**
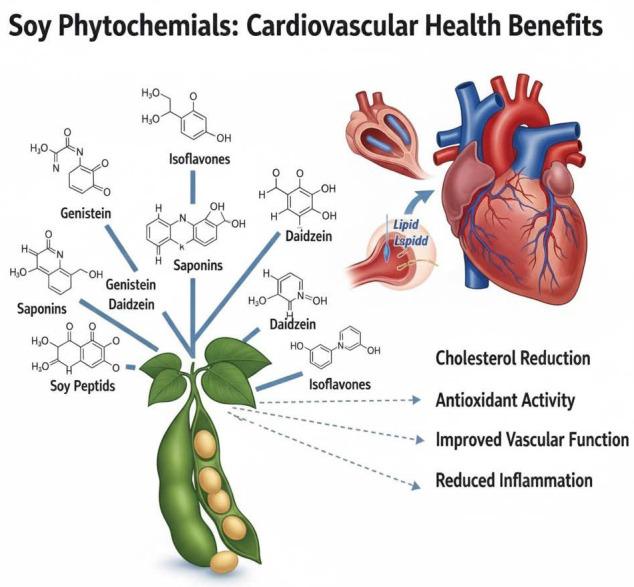
Cardiovascular health benefits through the phytochemicals of soybeans.

Soybeans have the most abundant phytochemicals, including isoflavones, saponins, phytosterols, phenolic acids, and phytic acid. Among these, isoflavones, especially genistein, daidzein, and glycitein, have received the most attention, being structurally like 17-β-estradiol, thereby exerting estrogenic and anti-estrogenic effects in the human body [10]. Isoflavones are suspected to have a role in cardiovascular risk reduction, increased bone density, alleviation of menopausal symptoms, and modulation of hormone-related cancers such as breast and prostate cancer [43] (Figure 2).

The second group of soy is saponins, which have antioxidant properties, cholesterol-lowering properties, and immune-modulating properties [43]. Phytosterols, especially β-sitosterol and campesterol, interfere with the intestinal absorption of dietary cholesterol, thereby improving lipid profiles [44]. Some phenolic acids like ferulic acid and caffeic acid have strong antioxidant potential that can scavenge free radicals and thus reduce oxidative stress. Phytic acid is generally considered an anti-nutrient; due to its mineral-binding capacity, it has also been postulated to provide antioxidant protection and hold promise for cancer prevention [44].

Bioavailability and concentration of these phytochemicals can be influenced by soybean genotype, cultivation conditions, and processing methods [45]. Fermentation significantly enhances the bioactivity of some phytochemicals, primarily isoflavones, when they are converted into aglycone forms that are more readily absorbed in the human gut [46]. Synergistic interactions among these compounds support the notion that soybeans can serve as a functional food with therapeutic potential for addressing metabolic, inflammatory, and degenerative diseases. Therefore, the phytochemical profile of soybean represents an important target for dietary intervention, nutraceutical development, and future clinical studies in disease prevention and health promotion [47].

## 4. Major Protein Fractions in Soybean: Glycinin (11S) and β-Conglycinin (7S)

Soybeans (*Glycine max*) yield a remarkable chemistry of phytochemicals, including isoflavones, saponins, phenolic acids, and peptides, significantly contributing to their antioxidant potential. Among these, the most studied are isoflavones such as genistein, daidzein, and glycitein. These compounds scavenge free radicals by donating hydrogen atoms or electrons to neutralize reactive oxygen species (ROS), thereby decreasing oxidative stress and cellular damage [48]. In vitro, soy isoflavones have DPPH radical scavenging activity, with IC_50_ values between 25 and 60 µM for different compounds and assay conditions [49].

Fermented soy products such as natto and miso have shown even greater antioxidant activity than nonfermented soy. The microbial enzymes facilitate the biotransformation of isoflavone glycosides, which are poorly bioavailable, into more bioavailable aglycones [50]. Furthermore, soy-derived bioactive peptides, released during enzymatic hydrolysis or digestion, are reported to possess oxygen radical absorbance capacity (ORAC), indicating strong antioxidant potential. These peptides can modulate antioxidant enzyme expression, such as superoxide dismutase (SOD), catalase (CAT), and glutathione peroxidase (GPx), enhancing the endogenous defense system against oxidative stress [50]. Clinical trials showed that continuous consumption of soy isoflavones (40–80 mg/day) significantly decreases oxidative stress markers such as malondialdehyde (MDA) and 8-OHdG in healthy and diseased populations [51]. Following the definitions given by FAO in 2013, soy protein has been widely accepted for its merit and value due to ~1.0 PDCAAS, comparable to that for casein and egg protein, but higher than that for most legume or cereal proteins. For example, pea protein has PDCAAS values of 0.69–0.82, and fava bean is around 0.75, mainly due to limitations in sulfur-containing amino acids like methionine and cysteine. In contrast, animal proteins such as milk casein, whey, and egg proteins generally score a PDCAAS of 1.0, indicating the presence of all amino acids in the human diet. The Digestible Indispensable Amino Acid Score (DIAAS) is a more modern method by which soy protein is superior to pea (DIAAS 70–80) and fava (DIAAS 65–75), but one score below milk whey (>100). Such comparisons underscore the unique status that soy holds as a sustainable plant-based protein source with an amino acid profile nearer to that of animal proteins; fava and pea protein, on the other hand, require complementary strategies to improve their protein quality [52].

## 5. Amino Acid Profile and Biological Value of Soy Protein

Soy protein is a nutritionally complete plant-based protein source, notable for its favorable amino acid profile and relatively high biological value (BV) among legumes. It provides all nine essential amino acids (EAAs) required for human nutrition, including lysine, leucine, isoleucine, valine, threonine, tryptophan, methionine, phenylalanine, and histidine [53]. Although methionine and cysteine (the sulfur-containing amino acids) are present in comparatively lower quantities, soy protein exhibits a high lysine content (~6.4 g/100 g protein), which complements cereal-based diets typically deficient in this amino acid [54]. According to FAO/WHO/UNU scoring patterns, the Protein Digestibility-Corrected Amino Acid Score (PDCAAS) of soy protein isolate approaches 1.0, indicating high digestibility and sufficient EAA composition for human health maintenance [55].

The biological value of soy protein, defined as the proportion of absorbed protein that becomes incorporated into body proteins, is approximately 74–85, lower than animal-derived proteins (e.g., egg or whey), but superior to most plant proteins [56]. Net Protein Utilization (NPU) and Digestible Indispensable Amino Acid Score (DIAAS) have also been used as indicators of soy protein quality. DIAAS values for soy protein isolates have been reported in the range of 84–90, depending on the processing conditions and degree of dehulling [57]. Enzymatic hydrolysis and fermentation can enhance amino acid availability and reduce anti-nutritional factors such as trypsin inhibitors and phytates, which otherwise impair amino acid absorption [58].

Soy protein’s high content of branched-chain amino acids (BCAAs), particularly leucine, supports muscle protein synthesis (MPS). However, its anabolic response is modestly lower compared to whey protein due to lower leucine bioavailability per gram [59]. Nonetheless, soy-derived peptides have demonstrated functional bioactivity, including antihypertensive, cholesterol-lowering, and antioxidant effects, further increasing their utility in clinical and sports nutrition. Therefore, soy protein serves as a sustainable, hypoallergenic, and functional alternative to animal proteins in both dietary and therapeutic applications [60].

The following table represents a comparative overview of the protein and energy density of selected protein sources, expressed per 100 g of dry weight. Among the listed items, whey (91 g) and casein (85 g) exhibit the highest protein concentrations, reflecting their role as purified dairy proteins commonly used in clinical and sports nutrition. Soybean (40 g) and fava beans (30 g) lead the plant-based category, offering substantial protein content alongside moderate caloric values, making them suitable for functional food formulations and dietary interventions targeting metabolic health. Cereals such as rice (7.5 g), millet (8.5 g), and teff (9.5 g) show lower protein density but contribute valuable calories (~350–360 kcal/100 g), supporting their role as staple energy sources. Quinoa (14 g) stands out among pseudocereals for its balanced amino acid profile and higher protein yield. Legumes like mung beans (24 g), chickpeas (21 g), and kidney beans (22 g) offer intermediate protein levels with favorable caloric profiles, reinforcing their utility in plant-forward dietary patterns. Overall, this table supports evidence-based selection of protein sources for nutritional planning, regulatory labeling, and functional food development, with values substantiated by peer-reviewed literature [61] (Table 2).

**Table 2:** Protein and calorie composition of cereals, beans, and other protein sources (g/100 g).

Food Items	Protein (g/100 g)	Calories (kcal/100 g)	Citation
Soybean	40	446	[62]
Fava beans	30	341	[63]
Whey	91	381	[64]
Casein	85	365	[65]
Rice	7.5	357	[66]
Quinoa	14	375	[67]
Teff	9.5	359	[68]
Millet	8.5	355	[69]
Mung beans	24	353	[70]
Chickpea	21	368	[71]
Kidney beans	22	340	[72]

## 6. Soy Isoflavones: Structure, Mechanism, and Therapeutic Potential

Soy isoflavones are a unique class of plant-derived polyphenolic compounds predominantly found in *Glycine max* (soybean). They belong to the flavonoid family and exhibit structural similarity to 17β-estradiol, enabling them to function as phytoestrogens [73]. The three primary isoflavones in soy are genistein, daidzein, and glycitein, typically present in glycoside forms genistin, daidzin, and glycitin, which are hydrolyzed in the gastrointestinal tract by β-glucosidase enzymes to yield their aglycone counterparts [74]. These aglycones are biologically active and capable of binding to estrogen receptors (ERα and ERβ), with a preferential affinity for ERβ. This selective binding underlies their selective estrogen receptor modulator (SERM)-like activity. Mechanistically, Genistein has been shown to modulate the PI3K/Akt, MAPK, and NF-κB signaling cascades, resulting in anti-inflammatory, antioxidative, antiproliferative, and pro-apoptotic effects [65]. Isoflavones also inhibit tyrosine kinases and topoisomerase II, which further contributes to their anticancer potential [66]. Additionally, daidzein can be metabolized by gut microbiota into equol, a more potent ERβ agonist, although only 30–50% of humans are “equol producers,” which may explain individual variability in therapeutic outcomes [67]. Clinically, soy isoflavones have demonstrated promise in reducing vasomotor symptoms in postmenopausal women, improving lipid profiles (by lowering LDL and raising HDL), and maintaining bone mineral density through enhanced osteoblastic activity and reduced osteoclastic resorption [68]. In oncology, genistein has shown potential in preventing hormone-dependent cancers, such as breast and prostate cancer, although results remain mixed in human trials due to differences in dosage, duration, and bioavailability [69]. In conclusion, soy isoflavones represent bioactive nutraceuticals with pleiotropic effects. Their therapeutic efficacy is influenced by molecular structure, metabolism, receptor affinity, and host gut microbiota composition, warranting further mechanistic and clinical investigations to establish standardized dietary recommendations [75].

## 7. Anti-Inflammatory Effects of Soy: Molecular Pathways

The anti-inflammatory properties of various present soy bio-actives, such as isoflavones (genistein, daidzein) and bioactive peptides, are mediated through diverse anti-inflammatory molecular signaling pathways [76]. Genistein downregulates pro-inflammatory gene expression via interference with the NF-κB (nuclear factor kappa-light-chain-enhancer of activated B cells) pathway, a vital control mechanism of inflammation. In vitro, genistein 10–50 µM concentrations significantly reduced LPS-induced expression of TNF-α, IL-1β, and IL-6 in macrophages [77]. Furthermore, soy isoflavones hinder LPS-induced phosphorylation of IκBα, followed by inhibition of NF-κB nuclear translocation, eventually leading to reduced cytokine production [78].

Another important pathway for soy bioactive modulation is that of MAPK (mitogen-activated protein kinase) signaling, especially ERK1/2 and p38 MAPK, which are responsible for cellular responses to inflammatory stimuli [79]. Soy protein hydrolysates have been shown to induce downregulation of these pathways in activated immune cells, resulting in decreased expression of COX-2 and inducible nitric oxide synthase (iNOS). Via activating Nrf2 (nuclear erythroid 2 p45-related factor 2), soy isoflavones could then induce the expression of heme oxygenase-1 (HO-1) and other cytoprotective enzymes, thereby offering a two-pronged antioxidant and anti-inflammatory strategy [80].

**Figure 3:**
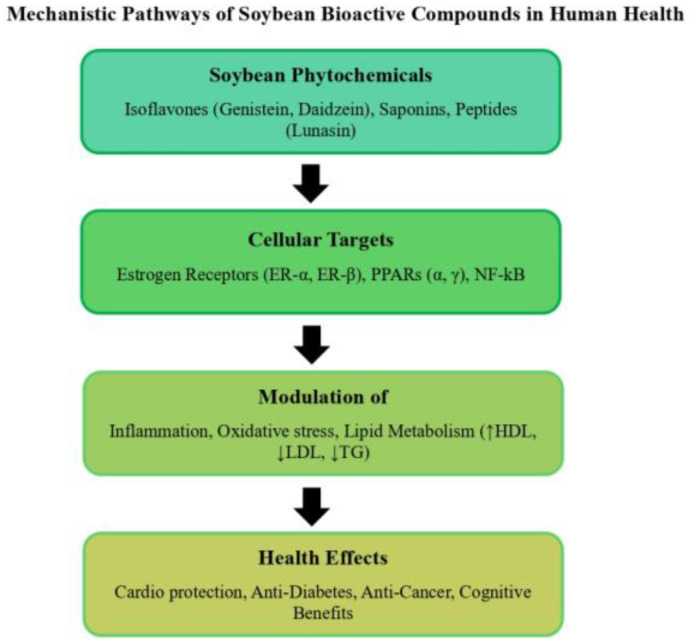
Mechanistic pathways of soybean bioactive compounds in human health.

**Figure 4:**
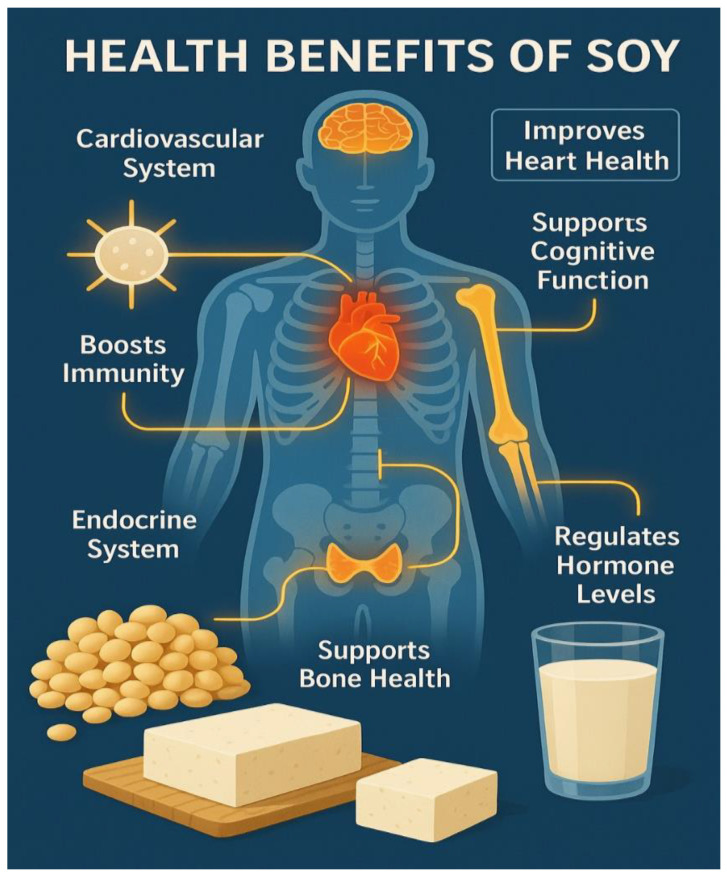
Various health benefits of soybeans.

In clinical settings, soy isoflavone supplementation (40–80 mg/day for 8–12 weeks) has been shown to significantly lower serum C-reactive protein (CRP) and interleukin-6 (IL-6) levels in patients with metabolic syndrome and rheumatoid arthritis [81]. These findings suggest that soy influences inflammation both transcriptionally and systemically. The anti-inflammatory action of soy proteins, by molecular mechanisms, involves inhibition of NF-κB and MAPK signaling, activation of Nrf2, and subsequent suppression of pro-inflammatory cytokines and enzymes [82] (Figure 3 and Figure 4).

## 8. Soybean in Cardiovascular Health: Lipid-Lowering and Atherogenic Effects

Regularly, soybean products, especially soy protein and soy isoflavones like genistein and daidzein, have been under research for a long time already because of their cardioprotective roles across the board, especially in lipid metabolism and the prevention of atherosclerosis [83]. Many clinical and mechanistic investigations provide evidence that it is possible to take soy protein and achieve significant reductions in serum total cholesterol, LDL-C (low-density lipoprotein cholesterol), and triglycerides with only slight increases in HDL-C (high-density lipoprotein cholesterol). The lowering of lipids emanating from soybeans is due to the amino acid composition, which is a higher arginine-to-lysine ratio, and the isoflavones influence the modulation of hepatic cholesterol homeostasis [84]. For instance, genistein has been shown to upregulate the expression of LDL receptors and inhibit the activity of HMG-CoA reductase, leading to a reduction in endogenous cholesterol synthesis [85]. In addition, soy peptides from protein hydrolysates were shown to have the capacity to inhibit micellar cholesterol solubility and lead to a decrease in cholesterol absorption intestine [86].

They exert anti-atherogenic effects through endothelial protection and anti-inflammatory mechanisms. This increases eNOS activity, resulting in improved vasodilation and reduced vascular stiffness. Genistein has been shown in vivo to reduce VCAM-1 and monocyte chemoattractant protein-1 (MCP-1), hence hampers monocyte recruitment and foam cell formation in the arterial wall [87]. Clinical trials indicate that regular soy consumption is associated with reduced carotid intima-media thickness (CIMT), an early marker of atherosclerosis, especially in postmenopausal women. These cumulative effects strongly position soy as a functional food with lipid-lowering, endothelial-protective, and anti-atherogenic properties [88]. The recommended amount of soy protein, as evidenced by research, to obtain cardiovascular benefit is close to 25 g per day. When incorporated into a diet that is low in saturated fat and cholesterol, this amount has an FDA-approved health claim that it can reduce the risk of heart disease by lowering total and LDL cholesterol. Specifically, for isoflavones, current studies associate a daily intake of about 100 mg of these compounds with a few cardiovascular gains, including blood pressure and endothelial-related improvements. Isoflavones are linked to a reduction in systolic blood pressure and improvement of artery health, while some meta-analyses attribute a 14–16% lower risk with an increase of 3 mg/day in isoflavone intake, for overall cardiovascular and coronary heart diseases. Soy protein: ~25 g/day for heart disease risk-reduction benefits. Isoflavones 100 mg/day are associated with improved cardiovascular markers and reduced risk. Benefits include cholesterol-lowering, blood pressure reduction, and improved vascular function. These doses are consistent with current dietary recommendations and clinical trials focusing on cardiovascular advantages in adults—that is, including postmenopausal women and persons with elevated cardiovascular risk [75].

## 9. Glycemic Control and Soy: Role in Diabetes Prevention and Management

Research in nutritional biochemistry in recent times has emphasized the role of soybeans as a probable food source for modulating glucose metabolism and preventing T2DM. Soybean is unique among plants in that it offers an array of isoflavones, peptides, and slow-acting carbohydrates that work together to maintain glycemic balance [89]. Soy isoflavones, namely genistein and daidzein, potentiate insulin signaling via PI3K/Akt and AMPK pathway activation, thus facilitating glucose uptake and utilization in skeletal muscle and liver tissues. In addition, these compounds were also found to stimulate GLUT4 translocation, thus enhancing insulin-mediated glucose clearance from plasma [90].

## 10. Soy and Hormonal Health: Estrogenic/Anti-Estrogenic Effects

Soy-derived isoflavones, particularly genistein and daidzein, have been extensively studied for their hormonal-modulating properties, owing to their structural similarity to 17β-estradiol. These phytoestrogens exhibit both estrogenic and anti-estrogenic activity by interacting with estrogen receptors (ERs), especially ERβ, with greater affinity than ERα [91]. This receptor selectivity is crucial in determining their tissue-specific effects, which vary according to physiological hormone levels, menopausal status, and microbiota composition [92]. In estrogen-deficient conditions, such as postmenopausal women, isoflavones function as weak estrogen agonists, alleviating vasomotor symptoms (e.g., hot flashes), improving bone mineral density, and enhancing lipid metabolism [93].

Critically, this extensive body of literature and randomized controlled trials has consistently shown that soy and isoflavones exert no negative effects on testosterone levels, sperm quality, or reproductive hormone profiles in men, thus reinforcing their endocrine safety. In addition, consumption of soy has no effects on menstrual cycle regularity or ovulatory function in women, further attesting to its safety in both sexes [93]. Together, soy isoflavones are a special category of bioactive substances capable of modulating hormonal effects by selective receptor binding and have considerable potential for the prevention of hormone-associated diseases and the alleviation of menopausal symptoms with no adverse reproductive effects [94].

## 11. Neuroprotective Effects of Soy and Cognitive Function

Cumulatively, these mechanisms emphasize the multidimensional neuroprotective potential of soy and suggest that soy food intake might be associated with long-term cognitive resilience, decreased vulnerability to age-related neurodegenerative disease, and healthful brain function in healthy and susceptible populations alike [95]. The age-related cognitive function improvement and neuroprotective activities of soy and soy bioactive phytochemicals have come under renewed scrutiny [96]. At the center of such activity are the soy isoflavones, genistein and daidzein, which possess antioxidant, anti-inflammatory, and estrogenic properties that modulate brain function. Phytochemicals can penetrate the blood–brain barrier to protect against oxidative stress, a major cause of neuronal damage and age-related cognitive decline, to maintain neuronal integrity [96]. By regulating oxidative enzymes, inhibiting lipid peroxidation, and enhancing endogenous antioxidant defenses, including superoxide dismutase and glutathione peroxidase, soy isoflavones may help to maintain neuronal integrity and synaptic plasticity [97].

Other mechanisms for the augmentation of cerebral blood flow and influence on neurotrophic factors like brain-derived neurotrophic factors (BDNFs) for maintenance of neurogenesis and synaptic transmission in such vital cognitive areas as the hippocampus are accessible to soy intake [98]. During estrogen-deficient states like menopause, isoflavones could serve as selective estrogen receptor modulators, mimicking estradiol effects on memory and learning while avoiding negative consequences of hormone replacement therapy [99]. Some neuroimaging and behavioral investigations have associated the regular use of soy with improved verbal memory, executive function, and processing speed in older adults, as well as a decrease in the risk of neurodegenerative illnesses [100]. At the cellular level, soy constituents may suppress neuroinflammation by inhibiting pro-inflammatory cytokines such as IL-6 and TNF-alpha and inhibiting microglial activation linked to the degeneration of nerve cells in Alzheimer’s and Parkinson’s diseases [101]. In addition, soy peptides may inhibit acetylcholinesterase, thus helping maintain steady levels of acetylcholine, an important mediator of cognition [102]. In addition, there is growing evidence of soy’s involvement in gut–brain axis modulation, thereby affecting neurotransmitter production and brain signaling pathways through its fermentation products [103]. There have been strong recent longitudinal studies and meta-analyses that indicated an association between the high consumption of soy products and a lesser reduction in risk of cognitive decline or neurocognitive disorders. The result of the dose–response meta-analysis, indicating the correlative effect on the consumption amount with the major neurocognitive disorders, showed that from a systematic review consisting of six studies with a population of 68,691, the increase of each 1 g/day of soy intake corresponded to an 8% decrease in the risk of developing the disorders. Fermented soy products like natto had stronger protection at 14% risk reduction for each 1 g/day rise. This was most pronounced among individuals who did not experience a history of stroke. However, the effects on mild cognitive impairment were inconsistent, and the results were somewhat different and heterogeneous. Another longitudinal study was conducted on elderly Japanese women, indicating a lowered risk of cognitive impairment in terms of total soybean and soy isoflavone intake as measured by the Mini-Mental State Examination (MMSE) [104]. Moreover, the meta-analysis with 16 randomized control trials showed a significant increase in the overall cognitive functioning and memory of adults who took soy isoflavones, thus offering further evidence of the cognitive benefits of soy. Dose–response effect: Each 1 g/day increase in soy intake decreased the risk of major neurocognitive disorder by 8%, with fermented soy providing stronger protection (14% risk reduction) [105].

## 12. Soy and Gut Microbiota: Probiotic Properties and Metabolomic Shifts

Soy consumption has been increasingly associated with positive modulation of gut microbiota composition, functioning as a dietary prebiotic due to its rich content of fermentable oligosaccharides, proteins, and polyphenolic compounds such as isoflavones [106]. Upon ingestion, non-digestible components of soy, particularly soy oligosaccharides (raffinose and stachyose), escape digestion in the upper gastrointestinal tract and are selectively utilized by beneficial gut microbes, including Bifidobacteria and Lactobacillus species [107]. This prebiotic effect leads to enhanced microbial diversity and the production of short-chain fatty acids (SCFAs), such as acetate, propionate, and butyrate, which play vital roles in maintaining colonic health, regulating inflammation, and modulating host metabolism [108]. Variability in equol producer status significantly impacts the health effects of soy since equol is more potent and bioavailable than its precursor, daidzein. Not all people can produce equol; this difference may influence the extent to which soy is able to improve menopausal symptoms, heart health, and possibly cancer risk. This factor should be emphasized in public health messages so that nutritional considerations can be personalized and so that consumers appreciate why soy effects may vary among individuals. The soybean plant contains isoflavones, mainly daidzein and genistein. Though these isoflavones have purported health effects, usually their effects depend on their conversion to active metabolites by gut bacteria, and equol is among the most important of these metabolites. Equol is preferred as the active form over daidzein for several reasons. It has a greater affinity for the estrogen receptor that mediates soy effects in the body. The half-life of equol in the body is longer than that of daidzein and hence has longer-lasting effects. Equol is a more efficient antioxidant than daidzein, thus protecting cells from damage. All these factors mean that people with certain gut bacteria which convert daidzein into equol (the so-called ‘equol producers’) may experience enhanced health benefits associated with the consumption of soy. The ability to produce equol varies considerably from population to population. In populations of Western descent, perhaps only 20–60% of individuals are equol producers, and high production levels may be found in some Asian populations. This seems to depend on the microbiota of the gut, which varies according to diet (e.g., vegetarians are more likely to be producers) and genetic predisposition [109].

The gut microbiota is also essential in the biotransformation of soy isoflavones into more bioactive metabolites such as equol and O-desmethylangolensin (ODMA) [104]. These metabolites exhibit higher estrogenic activity and improved bioavailability, which are associated with cardioprotective, anti-inflammatory, and anti-carcinogenic effects [110]. However, equol production is dependent on specific microbial populations that are present in only 30–50% of individuals, signifying interindividual variability in response to soy intake [111]. Studies using high-throughput sequencing and metabolomic profiling have revealed that regular soy consumption results in a metabolomic shift characterized by reduced levels of pathogenic bacteria (e.g., Clostridium perfringens) and increased abundance of SCFA-producing and mucin-degrading bacteria, contributing to enhanced intestinal barrier function and immune regulation [112]. The capacity of specific gut bacteria to metabolize the isoflavone daidzein into the more bioactive metabolite equol is one factor dependent on human beings. Roughly 30% of adults in Western populations are equol producers, while the prevalence is higher (around 50–60%) in Asian populations who regularly consume more soy. Equol producers may derive greater cardiovascular and hormone-dependent benefits from isoflavones. The composition of the gut microbiota, which may be affected by diet, antibiotics, etc., affects isoflavone bioavailability and its effects. From the view of differences in microbiota diversity and function, inter-individual differences could explain the variability in response to soy [113]. Epidemiological studies have shown that in breast cancer and other hormone-sensitive conditions, protection due to soy isoflavones is stronger in Asian populations as opposed to Western populations. These disparities could arise from genetic variations, early life exposure to soy, and differences in gut bacteria, leading to improved isoflavone metabolism. Timing of soy exposure (e.g., during childhood) and habitual dietary patterns in different ethnic groups influence the health effects observed. Genetic polymorphisms in enzymes involved in isoflavone metabolism may affect the efficacy and safety of isoflavone consumption [114]. Variants in genes regulating intestinal metabolism enzymes may influence individual responses and risk profiles. Host genetics also influences gut microbiome composition; a situation where the gut microbial composition can, in an indirect manner, affect isoflavone metabolism. In short, soy isoflavone effects can vary widely from person to person based on gut microbiota-dependent metabolism (especially equol production), ethnic dietary and genetic backgrounds, and timing of exposure, and hence are critical to determining who benefits from soy and isoflavone consumption [10].

## 13. Soy Intake in Adiposity Regulation and Metabolic Weight Control

Soy foods help regulate body weight through multiple physiological pathways, particularly in combating obesity and metabolic syndrome. Being rich in plant-based protein, isoflavones, and dietary fibers, they exert a satiating effect that aids appetite control and reduces overall caloric intake [115]. Moreover, bioactive peptides formed from the digestive soy protein may influence adipocyte differentiation, inhibit lipid accumulation, and promote lipolysis [116]. In addition to the above-mentioned mechanisms, soy isoflavones, such as genistein and daidzein, modulate lipid metabolism by interacting with important transcription factors like peroxisome proliferator-activated receptor gamma (PPAR-γ), which governs the formation of fat cells as well as insulin sensitivity [117].

Emerging evidence increasingly soy consumption reduces body fat mass, along with increases in lean body mass, especially when soy replaces animal protein [118]. Soy-enriched diets have been related to decreased serum levels of leptin, the adipokine that strongly relates to appetite control and fat oxidation, while having an increased serum adiponectin level in both human and animal studies [119]. Moreover, part of the low glycemic index property of soy is that it produces lower insulin spikes, often linked to weight gain and fat storage, thus contributing to better post-prandial glucose control [120].

However, beyond biochemistry, the impact of soy consumption on gut microbiota adds another argument in favor of weight management. Fermentable fibers and isoflavones in soy support the growth of microbial taxa associated with lean phenotypes and increased production of short-chain fatty acids (SCFAs), which enhance energy expenditure and improve metabolic profiles [121]. The multiple mechanisms discussed above collectively reinforce that the integration of soy into the diet serves to reduce the risk of obesity, enable the improvement of body composition, and facilitate the long-term maintenance of weight, together with the approaches of specific healthy eating patterns and lifestyle choices [122].

## 14. Nutraceutical Effects of Soy in Carcinogenesis Suppression Across Various Cancer Types

Soy and its bioactive constituents, as stated within the scope of modulation of risk and progression of malignancy, especially those that are hormone sensitive, such as breast and prostate cancer, and colorectal cancer [123]. Isoflavones, that is, genistein and daidzein, found in high amounts in soybeans, act as phytoestrogens in that they could bind to both estrogen receptors (ERα and ERβ), and thus bring about selective modulation of these receptor activities. This dual behavior as agonists and antagonists is believed to play a major role in how hormone-fed tumors, particularly ER+ breast cancers, grow [124]. The commerce in cancer cell proliferation inhibition, apoptosis induction, and angiogenesis inhibition by genistein has been demonstrated in both in vitro and in vivo models. In prostate cancer, soy isoflavones downregulate androgen receptor signaling and modulate enzymes involved in steroidogenesis, resulting in reduced tumor growth [125]. Epidemiological studies suggest that populations with higher lifelong soy intake, particularly in East Asia, exhibit significantly lower incidence and mortality rates of prostate and breast cancers, supporting the hypothesis that early and consistent exposure may confer protective effects. In the context of colorectal cancer, soy components have demonstrated the ability to modulate Wnt/β-catenin signaling, reduce inflammation in colonic mucosa, and alter gut microbial metabolites that influence carcinogenesis [126].

**Figure 5:**
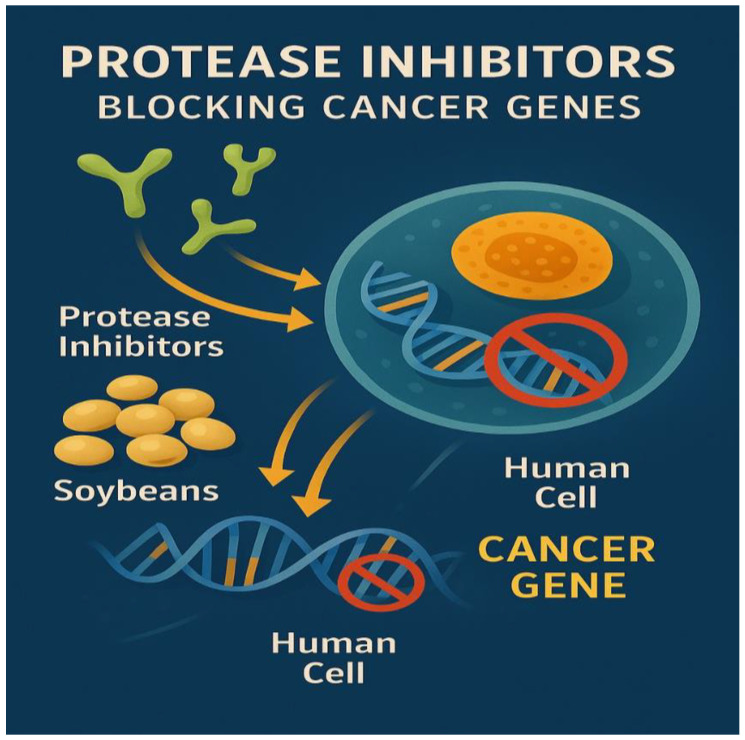
Protease inhibitors blocking cancer genes.

Furthermore, soy peptides generated during digestion exhibit antioxidant activity, reduce oxidative stress, and can influence detoxification pathways by modulating phase I and phase II liver enzymes involved in carcinogen metabolism [127]. The fermentation of soy foods also enhances isoflavone bioavailability, further amplifying their chemopreventive potential. While human studies yield mixed findings largely due to differences in isoflavone metabolism and individual gut microbiota, the preclinical and epidemiological data collectively indicate that soy-based dietary interventions may play a supportive role in cancer prevention, particularly when introduced as part of a balanced, plant-forward dietary pattern [128] (Figure 5).

## 15. Functional and Fortified Soy-Based Products

In recent years, soy-derived ingredients have emerged as central components in the development of functional and fortified foods aimed at addressing global nutritional challenges. Due to their high-quality protein content, abundance of isoflavones, saponins, and essential fatty acids, soy products are increasingly utilized in the formulation of health-enhancing food products [129]. Soy concentrations and protein isolates are extensively used in meat alternatives, dairy alternatives, bakery items fortified with protein, and beverages to impart not just nutritional but beneficial textural properties as well 118[]. Fortified soy milk, tofu, and yogurt with calcium, vitamin D, and B-complex vitamins are especially significant in high-risk populations such as vegans, vegetarians, and older adults [130]. Further, soy food ingredients have also been shown to contribute to satiety, decrease cholesterol, and control glycemia, thus making it possible to produce foods to control non-communicable diseases, including obesity, cardiovascular disease, and diabetes [131].

Technologically, soy proteins have good emulsifying, water retention, and gelation properties; therefore, they are ideally suited for application in processed and convenient foods [131]. Breakthroughs in newer processing technologies like extrusion, fermentation, and enzymatic hydrolysis have also facilitated the development of novel soy food products with improved bioavailability and sensory acceptability [132]. Fermented soy foods such as tempeh, miso, and natto provide probiotic and prebiotic activities, enhancing gut well-being and immune modulation. Soy flour and soy peptides are being utilized in snack bars, fortified cereals, infant formula, and clinical and industrial nutritional supplements [133]. Soy’s renewability and versatility, along with its established health benefits, highlight its central position in future-oriented functional foods for preventive and therapeutic nutrition.

## 16. Biotechnological Advances in Soybean Breeding and Biofortification

New biotechnological tools have transformed soybean breeding to enhance its nutritional quality and agronomic traits. Marker-assisted selection (MAS), CRISPR-Cas9 genome editing, and transgenic strategies have enabled the targeted modification of genes controlling important traits such as protein content, amino acid profile, isoflavone level, and biotic and abiotic stress resistance [134]. The coupling of genomic selection and high-throughput phenotyping has pushed the discovery of high-yielding elite lines with increased bioactive compounds like β-conglycinin and glycinin being the determinants for health-enhancing activity. Biofortification has also aimed to enrich vital micronutrients, such as iron, zinc, and folate, in soybean seeds to counteract unexpected hunger among vulnerable populations [135]. Furthermore, metabolic engineering has also facilitated the alteration of fatty acid content to decrease the saturated fats and improve the content of healthy unsaturated fatty acids such as oleic acids. These technologies not only enhance the functional quality of soybeans for food and nutraceutical uses but also promote sustainable agriculture by producing high-yielding, nutrient-rich cultivars with improved vigor [136]. Overall, contemporary biotechnological techniques continue to propel soybean variety improvement for health and environmental purposes, representing a critical shift towards precision nutrition and food security [136]. Processing technologies over the years have influenced the bioavailability and health effects of bioactive agents from soy, especially isoflavones. These changes in the bioavailability of compounds are mainly due to chemical changes and inactivation of the anti-nutritional factors usually found in raw soy. The difference is most prominent between fermentation and any other heat-based processes, such as extrusion.

Fermentation: The Gold Standard for Bioavailability: Fermentation is a process that maximizes the health benefits of soy on bioavailability through the release of the most relevant bioactive compounds. In unprocessed or raw soybeans, isoflavones are almost entirely in glycoside compounds—or bound to a sugar molecule—and the human gut has little capability to absorb such large molecules [137].

Mechanism: Fermentation, using microorganisms (like the bacterium in miso or the mold in tempeh) in the production of the enzyme β-glucosidase, activates the reaction that cleaves off the isoflavone from its sugar group, thus transforming the less-bioavailable glycosides (e.g., daidzin, genistin) into highly absorbable aglycones (e.g., daidzein, genistein).Health Impact: The conversion gives rise to a quicker and better absorption of isoflavones into the bloodstream. Hence, fermented soy products such as miso, tempeh, and natto are often associated with greater health benefits, such as stronger antioxidant capacity and greater advantage for heart health and menopausal symptoms. Fermentation also adds probiotics beneficial to the body and may reduce some anti-nutritional factors, including trypsin inhibitors that interfere with protein digestion [138].

Heat Processing and Extrusion: A Mixed Bag. Heat processing, cooking, or roasting, and high-temperature short-time processes, such as extrusion (used in the production of textured vegetable protein, or TVP), yield a more complex effect. Positive Impact: Heat processing is also important in inactivating anti-nutritional factors, for instance, the destruction of trypsin inhibitors that would otherwise hinder the digestion and absorption of soya protein. This is a critical step that aids in the easier digestion of soy foods [139].

Negative Impact: However, overdoing the heat can also lead to degradation or loss of some isoflavones. Studies show that heat can enhance the proportion of aglycones by releasing them from the cellular matrix; however, studies have also reported that showed an overall decrease in total isoflavone content. The effects vary depending on the specific method, temperature, and duration of the heat treatment. Health Impact: The allergens contained in products produced under intense heat, such as some soy protein isolates or extruded products, are probably less this way than if viewed as such raw. This may mitigate some of the health impacts attributed to the isoflavones, even while enhancing protein digestibility [140].

Other Processing Methods, Isolation, and Concentration: Soy protein isolates and concentrates were prepared using many processing methods in most processed food products or protein powders, and generally involved methods that significantly reduce isoflavone content. Isoflavones would be washed from this product as water or alcohol solubilizes them during extraction, and would give a product high in protein but low in bio-actives. The processing of soybeans is a critical factor in determining the health profile of the final product. Basic heat treatment is essential for increasing the digestibility of protein, but fermentation stands supreme among processing technologies for improving the bioavailability and health impact of soy isoflavones. Thus, traditional fermented foods from soybeans are regarded as most beneficial when considering maximizing the advantageous effects of these important plant compounds [141].

## 17. Diagnostic Innovations and Allergen Mitigation of Soy Protein

Soy allergy is an IgE-mediated hypersensitivity reaction against certain allergenic proteins present in soybean, Gly m 5 (β-conglycinin) and Gly m 6 (glycinin) [142]. These are thermally stable and resistant to digestion and are thus highly potent allergens with the ability to provoke mild to severe reactions like urticaria, gastrointestinal disturbance, respiratory difficulty, or even anaphylaxis in sensitized individuals [143]. Diagnosis generally depends on a combination of patient history, skin prick testing (SPT), measurement of serum-specific IgE, and oral food challenge, which continue to be the gold standard for the diagnosis of clinical allergy. Newer diagnostic reagents like component-resolved diagnostics (CRD) improve sensitivity by identifying individual allergen components instead of whole protein extracts [144]. Treatment is based on strict soy and soy product avoidance and patient education on the concealed use of soy in processed foods. Epinephrine injectors are provided to susceptible patients. Immunotherapeutic treatments such as oral immunotherapy (OIT) and sublingual immunotherapy (SLIT) have been a promising area in the recent past for desensitizing allergic individuals, though investigational for soy [145]. Food labeling law is an important milestone in the safeguarding of consumers via compulsory labeling of foods with significant allergens, such as soy, on packaged foods. Ongoing monitoring and public education are necessary to decrease the soy allergy burden and enhance patient safety [146].

## 18. Sustainability of Soy Production and Its Environmental Impact

Soybean production is a significant contributor to the world’s food, feed, and industrial markets, but its rapid global growth worries environmental sustainability [147]. Being a legume, soybean also naturally fixes nitrogen and therefore enhances the fertility of the soil by having less reliance on artificial nitrogen fertilizers, which lessens greenhouse gas emissions [148]. Yet, extensive cultivation of monoculture on a large scale in nations such as Brazil, Argentina, and the U.S. has resulted in widespread deforestation, reduction in ecosystem diversity, and depletion of water, primarily in vulnerable regions such as the Amazon basin [149]. Soybean-based land-use change is recognized as a major contributor to the carbon footprint and fragmentation of habitat by life cycle assessments. To overcome these, eco-friendly farming practices like conservation tillage, integrated pest management, crop rotation, and agroforestry are being promoted [149]. In addition, precision agriculture technology, remote sensing, and gene-edited soybean cultivars with increased yield and abiotic stress resistance are enhancing the productivity of resource use. Certified sustainable soy programs (e.g., RTRS and ProTerra) offer evidence of green practices and supply chain traceability [150]. In addition, the expanding market for plant-based food is changing demand from feed-grade to food-grade soy, which could alleviate ecological stress. Soy continues to be a nutrient-dense protein in world nutrition, but future sustainability will hinge on synergistic action among environmental policy, land stewardship, and technology-based agroecosystems.

## 19. Future Directions: Integrating Soy in Global Food and Health Policies

The growing recognition of soy as a nutritionally dense, environmentally sustainable, and functionally versatile crop positions it as a key candidate in future global food and health policies. With its high-quality protein content, essential amino acids, and bioactive components such as isoflavones, soy is increasingly being explored in strategies to address protein-energy malnutrition, reduce the burden of non-communicable diseases (NCDs), and support healthy aging. Global organizations like the FAO and WHO have acknowledged plant-based diets, including soy, as pivotal in achieving the Sustainable Development Goals (SDGs), particularly those related to zero hunger, good health and well-being, and responsible consumption. Future policy measures will demand high-priority placement of soy in school lunches, clinical dietary recommendations, buying consumer food, and public feeding to support nutritional equity. Additionally, soy’s low environmental impact in comparison to animal proteins is complemented by its action on climate-resilient agriculture, positioning soy as a shared crop for climate change adaptation initiatives. Integration at the policy level will also include inputs on research, infrastructure, and training to support safety, allergen control, and cultural acceptance. Interagency collaborations among governments, educational institutions, and the private sector will be critical in large-scale sustainable soy production and in continuing to build its importance in public health nutrition. As food systems transition toward plant-forward models, soy is poised to become a cornerstone of inclusive, health-driven, and ecologically responsible food policies worldwide.

## 20. Conclusions

Soybean is a highly nutritious legume, providing a comprehensive amino acid profile and functional bioactive compounds that are advantageous for human health. Protein-energy malnutrition can be considerably reduced by including it in the diet, especially in developing nations like Pakistan. Tofu, tempeh, soy flour, soy milk, and soy protein isolate (SPI) are just a few of the soy-based products that have become increasingly popular worldwide. Because of its high protein content, adaptability, and use in both everyday and medicinal meals, soy protein isolate is the most popular of these. Soy-based treatments can improve nutritional quality and lessen nutrient deficits in vulnerable individuals in areas with food insecurity. Nonetheless, soy allergies are a problem, particularly in kids, and can result in symptoms that range from minor skin reactions to life-threatening anaphylaxis. Oral meal challenges, skin prick testing, and serum-specific IgE assays are examples of diagnostic procedures. The main way to manage the allergy is to avoid certain foods, but most kids outgrow it as they become older. Promising options are provided by research into hydrolyzed soy proteins and low-allergen soy cultivars. In general, soy products offer a sustainable way to enhance world nutrition and lessen the burden of malnutrition and associated illnesses when used responsibly. This present systematic review strictly follows PRISMA guidelines and details the biochemical, physiological, and nutraceutical effects of soybean consumption across all revelations published during 2018–2025. As a result, the cumulatively obtained information undoubtedly establishes soybean as a bioactive-dense legume, characterized by its two major storage proteins, glycinin (11S) and β-conglycinin (7S), and a complete amino acid profile giving it a high biological value. Isoflavones, the most dominant phytoestrogens, produce pleiotropic effects by both estrogen receptor-dependent and -independent pathways, including modulations of lipid metabolism, glycemic homeostasis, and inflammatory cascades, antiproliferative, and pro-apoptotic effects in carcinogenesis models. From evidence obtained through randomized controlled trials, statistically significant and clinically relevant decreases in serum LDL-C, improvement in endothelial function, and decreases in pro-inflammatory markers, soy protein, and isoflavones have thus far verified the cardiometabolic benefits of intake of these constituents. Moreover, neuroprotective, microbiota-modulating, and weight-regulating properties revealed by the data go beyond cardiometabolic implications. It is interesting to note that metabolomic studies show that equol production capacity substantially mediates inter-individual variability concerning the endocrine and metabolic responses. Finally, breeding strategies, biofortification, and allergenicity mitigation are extending the safety and applicability of soy use among diverse populations in the world, and soy-based functional and fortified preparations offer translational potential for targeted use in dietary interventions. Most significantly, there is insufficiently consistent adverse-effect outcome information with respect to thyroid function or reproductive hormones; hence, it strengthens the available evidence base supporting the general safety of soy for consuming adults. Future research should emphasize multi-omic approaches, dose–response meta-analyses, and longitudinal cohort studies that could be made to elucidate causal pathways, gene-diet interactions, and the long-term clinical impact of soy consumption on the trajectories of non-communicable disease. Importantly, the inclusion of soy in national dietary guidelines, along with government-supported school feeding programs and sustainable agri-food systems, represents a major chance to counteract the current protein shortage around the globe and contribute directly to planetary health. All in all, soybeans emerge as a dietary constituent and a bio-functional system capable of harmonizing molecular nutrition, chronic disease prevention, and environmental resilience.

## Data Availability

No new data were created or analyzed in this study.
